# Automatic differentiation of Grade I and II meningiomas on magnetic resonance image using an asymmetric convolutional neural network

**DOI:** 10.1038/s41598-022-07859-0

**Published:** 2022-03-09

**Authors:** April Vassantachart, Yufeng Cao, Michael Gribble, Samuel Guzman, Jason C. Ye, Kyle Hurth, Anna Mathew, Gabriel Zada, Zhaoyang Fan, Eric L. Chang, Wensha Yang

**Affiliations:** 1 Department of Radiation Oncology, LAC+USC Medical Center, Los Angeles, CA USA; 2 Department of Radiation Oncology, Medical Physics Division, Norris Cancer Hospital, Keck School of Medicine, University of Southern California, 1441 Eastlake Ave., Los Angeles, CA 90033 USA; 3 Keck School of Medicine, University of Southern California, Los Angeles, CA USA; 4 Department of Pathology, Keck School of Medicine, University of Southern California, Los Angeles, CA USA; 5 Department of Neurological Surgery, Keck School of Medicine, University of Southern California, Los Angeles, CA USA; 6 Department of Radiology, Keck School of Medicine, University of Southern California, Los Angeles, CA USA

**Keywords:** Translational research, Cancer imaging

## Abstract

The Grade of meningioma has significant implications for selecting treatment regimens ranging from observation to surgical resection with adjuvant radiation. For most patients, meningiomas are diagnosed radiologically, and Grade is not determined unless a surgical procedure is performed. The goal of this study is to train a novel auto-classification network to determine Grade I and II meningiomas using T1-contrast enhancing (T1-CE) and T2-Fluid attenuated inversion recovery (FLAIR) magnetic resonance (MR) images. Ninety-six consecutive treatment naïve patients with pre-operative T1-CE and T2-FLAIR MR images and subsequent pathologically diagnosed intracranial meningiomas were evaluated. Delineation of meningiomas was completed on both MR images. A novel asymmetric 3D convolutional neural network (CNN) architecture was constructed with two encoding paths based on T1-CE and T2-FLAIR. Each path used the same 3 × 3 × 3 kernel with different filters to weigh the spatial features of each sequence separately. Final model performance was assessed by tenfold cross-validation. Of the 96 patients, 55 (57%) were pathologically classified as Grade I and 41 (43%) as Grade II meningiomas. Optimization of our model led to a filter weighting of 18:2 between the T1-CE and T2-FLAIR MR image paths. 86 (90%) patients were classified correctly, and 10 (10%) were misclassified based on their pre-operative MRs with a model sensitivity of 0.85 and specificity of 0.93. Among the misclassified, 4 were Grade I, and 6 were Grade II. The model is robust to tumor locations and sizes. A novel asymmetric CNN with two differently weighted encoding paths was developed for successful automated meningioma grade classification. Our model outperforms CNN using a single path for single or multimodal MR-based classification.

## Introduction

Meningiomas are the most common primary intracranial tumors and account for 38% of all primary brain tumors, with an estimated 34,210 new cases projected to occur in the United States in 2020^1^. Meningiomas are further classified into world health organization (WHO) Grade I, II, and III, which account for 80.5%, 17.7%, and 1.7% of meningiomas, respectively^2^. However, only 40% of meningiomas are histologically confirmed, with the remaining diagnosed radiologically^1^. The natural progression and recurrence rates vary by WHO grade with a 5-year recurrence rate after gross total resection (GTR) of 7–23% in Grade I meningiomas and 50–55% in Grade II and 72–78% in Grade III meningiomas^1^. With a wide discrepancy in the natural course of meningiomas, the importance of Grade based on radiologic findings is paramount as the initial decision of whether or not to resect a meningioma may be based solely on its radiologic findings, and recommended treatment can range from observation to surgical resection with adjuvant radiation^3–5^.

Computer-aided diagnosis (CAD) has become a growing focus in medical imaging and diagnostic radiology^6^. Significantly improved performance has been reported for solving complex problems, including image colorization, classification, segmentation, and pattern detection using deep learning. Among these methods, convolutional neural networks (CNN's) have been extensively studied and shown to improve prediction performance using large amounts of pre-labeled data^7–10^. Specifically, CNN's have outperformed conventional methods successfully, particularly in medical image classification^11,12^. With the help of deep learning, radiological grading of meningiomas can guide treatment options that include resection, radiation, or observation, particularly in patients who do not undergo pathologic diagnosis. Nonetheless, relatively few imaging studies have differentiated meningioma grade and the few that have do not combine MR sequences. Two prior studies used deep learning radiomic models to predict Grade based on T1-CE MR alone and achieved accuracy rates of 0.81 and 0.82^13,14^. Because multiparametric MR images are commonly acquired to assist brain tumor diagnosis and classification, it is reasonable to hypothesize that some MR sequences or a combination of multiple MR sequences may be better suited for automated meningioma grading. Banzato et al. separately tested two networks on T1-CE MR images and apparent diffusion coefficient (ADC) maps for meningioma classification and achieved the best performance of grade prediction with an accuracy of 0.93 using Inception deep CNN on ADC maps^15^. However, different MR imaging sequences were not combined to improve performance further. Our study sought to use T1-CE and T2-FLAIR, two of the most commonly used MR sequences for diagnosis, with a layer-level fusion^16^ of symmetric weightings to predict meningioma grade. Moreover, motivated by the success of asymmetric learning from two different kernels^17^, an asymmetric learning architecture from multimodal MR images was built using an additional path to predict meningioma grades.

## Methods and materials

### Subjects and MR images

The study was approved by the institutional review board (IRB) to review human subjects research (protocol ID HS-18-00261) at the University of Southern California. All methods to acquire image data were performed following the relevant guidelines and regulations. Informed consent has been obtained at the time of initial patient registration. The study included 96 consecutive treatment naïve patients with intracranial meningiomas treated with surgical resection from 2010 to 2019. All patients had pre-operative T1-CE and T2-FLAIR MR images with subsequent subtotal or gross total resection of pathologically confirmed Grade I or grade II meningiomas. Grade III meningiomas were excluded due to their rare occurrence, not allowing for a robust dataset. A neuropathology team reviewed histopathology, including two subspecialty trained neuropathologists and one neuropathology fellow. The meningioma grade was confirmed based on current classification guidelines, most recently described in the 2016 WHO Bluebook^18^.

MR scanners from different vendors were used. Those include 3 T or 1.5 T GE (Optima™, Discovery™, Signa™), Philips (Achieva™, Intera™), Toshiba (Titan™, MRT 200™, Galan™), Hitachi (Oasis™, Echelon™), and Siemens (Symphony™, Skyra™). The scanning parameters for the T1-CE sequence include TR of 7–8 ms, TE of 2–3 ms, the in-plane volumetric dimension of 256 × 256, the isotropic in-plane spatial resolution of 1.016 mm, slice thickness of 1–2 mm, FOV of 100 mm^2^ and flip angle of 15°. The scanning parameters for the T2-FLAIR include TR of 8000–11,000 ms, TE of 120–159 ms, in-plane volumetric dimension 256–512 × 256–512, isotropic in-plane resolution of 0.4–0.9 mm, slice thickness of 2–7 mm, FOV of 80–100 mm^2^ and flip angle of 90°–180°.

### MR pre-processing

**Figure 1:**
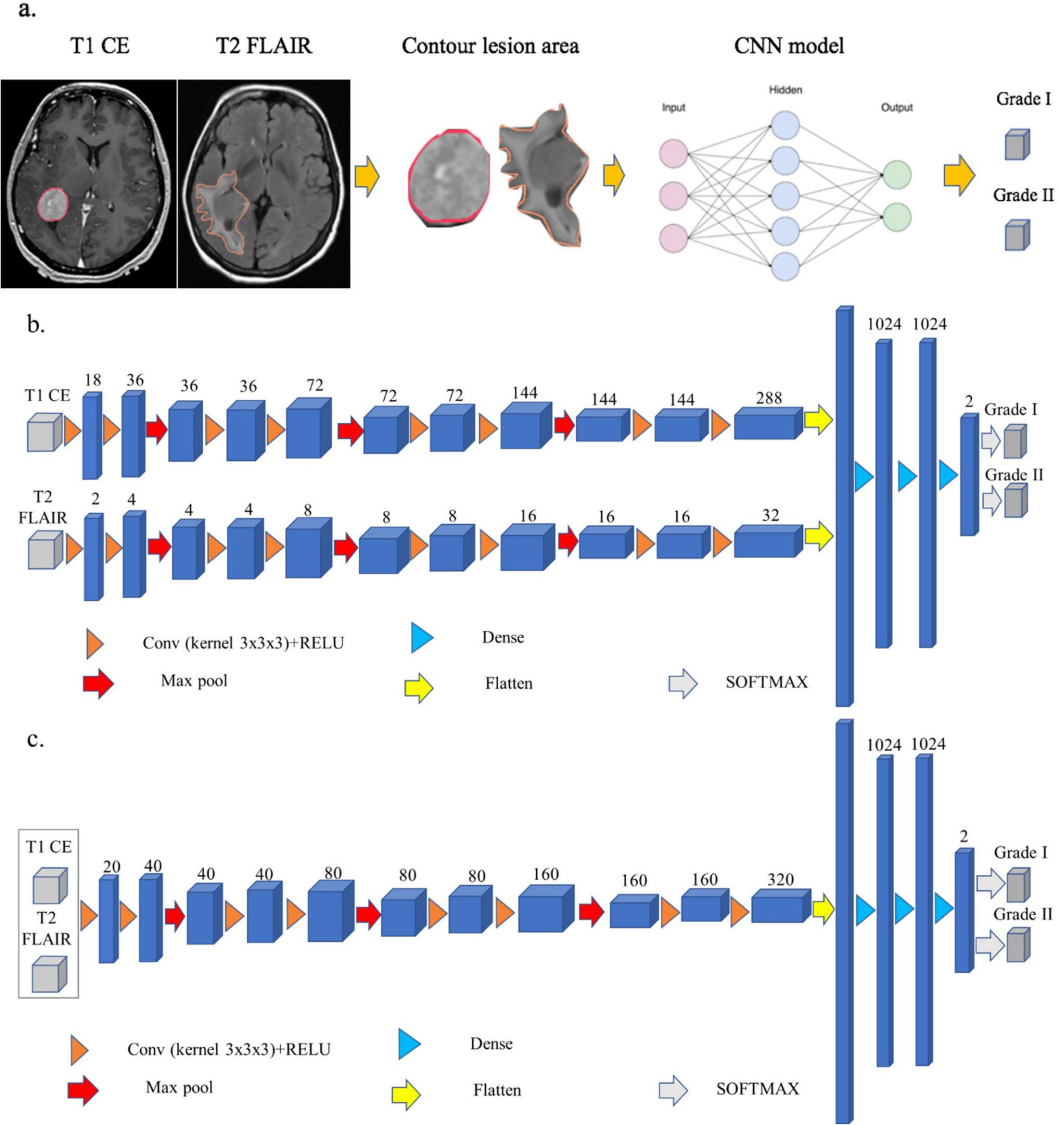
(**a**) Workflow used for this study. (**b**) An illustration of the architecture of our novel convolutional neural network. (**c**) An illustration of the architecture of the traditional convolutional neural network. Each blue cuboid corresponds to feature maps. The number of channels is denoted on the top of each cuboid.

The image processing workflow is shown in Fig. 1a. DICOM files containing T1-CE and T2-FLAIR were exported to VelocityAI™ (Varian, Palo Alto, CA). The T1-CE and FLAIR MR images for each patient were acquired in the same study with the patient holding still on the MR table. They have original DICOM registration with anatomy aligned, assuming the patient did not move unintentionally between T1-CE and FLAIR acquisitions. We double-checked the initial DICOM registration by overlaying the two images and inspecting the alignment of the skull and ventricles. If the alignment is off, we then perform an automatic rigid registration using the mutual information algorithm installed on VelocityAI™. T2-FLAIR was then resampled to T1-CE. The hyperintense T1-CE tumor and hyperintense T2-FLAIR and tumor were manually contoured on each image. The organized dataset, including original MR images, was resampled to an isotropic resolution of 1 × 1 × 1 mm^3^. The tumor contours were then exported to a LINUX computational server, equipped with 4 × RTX 2080 Ti 11 GB GPU and Devbox (X299, I9-7900 ×, 16GB × 4, 1 × 240 GB OS SSD, 1 × 10 TB DATA HDD, Ubuntu 16.04) for model training and testing.

### Cross-validation procedure

The random rotation was used for data augmentation in cross-validation. Final model performance was assessed by tenfold cross-validation^19^, in which each fold consisted of randomly selected 10 testing, 76 training, and 10 validation subjects.

### Network architecture

As shown in Fig. 1b, a novel 3D asymmetric CNN architecture with two encoding paths was built to capture the image features based on the two MR sequences (T1-CE and T2-FLAIR). Each encoding path had the same 3 × 3 × 3 kernel with a different number of filters to control the unique feature of each MR sequence. The asymmetric CNN implements an 18:2 ratio for the T1-CE vs. T2-FLAIR encoding paths. Such a network can learn the individual feature representation from the two MR sequences with higher weighting on the T1-CE while incorporating features from T2-FLAIR. Inside each encoding path, the corresponding kernel convolution was applied twice with a rectified linear unit (RELU), a dropout layer between the convolutions with a dropout rate of 0.3, and a 2 × 2 × 2 max-pooling operation in each layer^20^. The number of feature channels was doubled after the max-pooling operation. After feature extraction by 3D convolutional layers, three fully connected layers were applied to map 3D features. Dropout layers with a rate of 0.3 were applied after each fully connected layer. In the final step, the last fully connected layer was used to feed a softmax, which maps the feature vector to binary classes. Cross-entropy was applied as the loss function. Glorot (Xavier) normal initializer was used for this asymmetric CNN^21^, which drew samples from a truncated normal distribution centered on zero with stdev = sqrt (2/(fan_in_ + fan_out_)). fan_in_ and fan_out_ were the numbers of input and output units, respectively, in the weight tensor. Adam optimizer was applied to train this model^22^. The learning rates ranging from 1 × 10^–6^ to 1 × 10^–4^ were tested; a learning rate of 2 × 10^–5^ with 1000 epochs was selected based on model convergence. A 3D symmetric CNN architecture with a 10:10 ratio for T1-CE vs. T2-FLAIR dual encoding paths was also constructed for comparison. The code can be reviewed and accessed on https://github.com/usc2021/YCWYAV.

To assess the model improvement from dual encoding paths, a traditional CNN (Fig. 1c) was also built with a single encoding path, inside which identical network parameters were used as the dual-path CNN architecture.

### Model performance evaluation

To evaluate the performance of this asymmetric CNN model, the following quantitative metrics were assessed with the pathologic Grade as the ground truth: accuracy, sensitivity, specificity, receiver-operating characteristic (ROC) curve analysis, and the area under the curve (AUC). True positive was defined as the number of pathologic grade II meningiomas correctly identified as Grade II.

To assess if different tumor volumes from T1-CE and T2-FLAIR will have an impact on the model performance, we define $R=\frac{{V}_{T1CE}}{{V}_{T2FLAIR}}$, where ${V}_{T1CE}$ is the tumor volume from T1-CE MR and ${V}_{T2FLAIR}$ is tumor volume from T2-FLAIR MR.

### Ethics approval

The study is approved by IRB.

### Consent for publication

The publisher has the authors' permission to publish.

## Results

**Figure 2:**
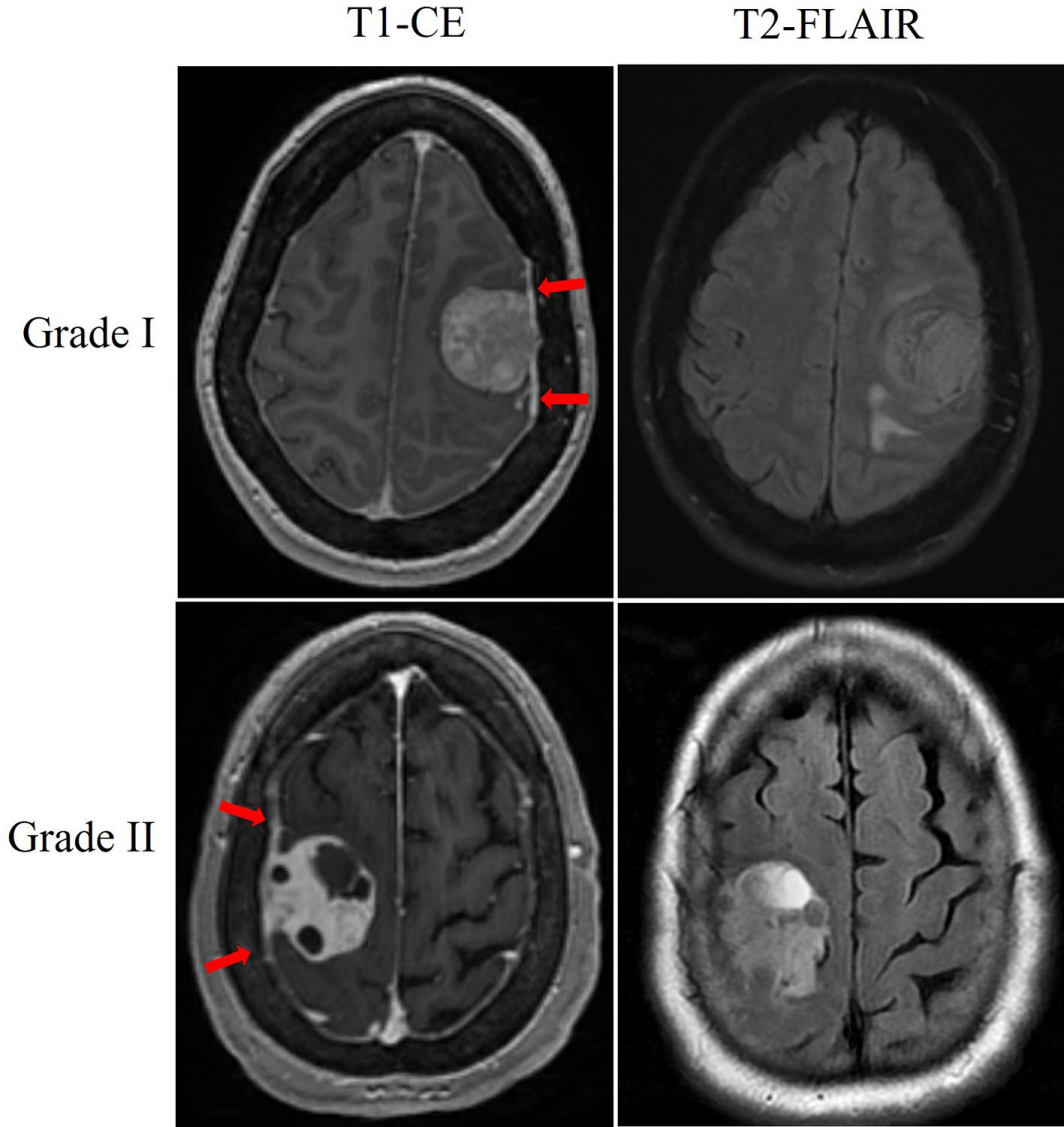
Meningioma with typical radiological signs for Grade I and Grade II. T1-CE MRI after gadolinium injection depicts heterogeneous contrast enhancement. The red arrows show the dural tail on T1-CE.

A total of 96 patients with pathologically diagnosed grade I or grade II meningiomas were evaluated. Of these patients, 55 (57%) were Grade I, and 41 (43%) were Grade II. The median age was 56 (25–88) and included 69.8% female and 30.2% male patients. Meningioma locations from more frequent to less frequent followed the order of anterior and middle cranial fossa (43), convexity (20), falx and parasagittal (18), posterior cranial fossa (13), and lateral ventricle (2). An example of typical Grade I and II meningioma radiologic findings such as T1 contrast enhancement, presence of a dural tail, and adjacent T2-FLAIR hyperenhancement are shown in Fig. 2.

To demonstrate the individual predictive performance for different MRI sequences, T1-CE and T2-FLAIR were trained by traditional single path CNN, respectively. With the traditional CNN, 69 (72%) patients were predicted correctly, and 27 (28%) were misclassified based on T1-CE imaging. If T2-FLAIR was applied to the traditional CNN model, 31 (32%) patients were predicted correctly, and 65 (68%) were misclassified. When both T1-CE and T2-FLAIR are combined in the single path CNN, an in-between performance was achieved, with 63 (66%) correct meningiomas classified and 33 (34%) misclassified.

Due to the low accuracy of the single path traditional model, a dual path fusion model was studied by extracting features from the sequences of T1-CE and T2-FLAIR, respectively. Using the symmetric CNN model, 75 (78%) patients were classified correctly and 21 (22%) were misclassified based on their pre-operative imaging, with a filter ratio of 10:10 for T1-CE and T2-FLAIR. The misclassified meningiomas included 10 grade II and 11 grade I meningiomas. However, applying an asymmetric model with a filter ratio of 18:2 for T1-CE and T2-FLAIR achieved a correct prediction of 86 (90%) meningiomas and misclassification of 10 (10%) meningiomas. The misclassified meningiomas included 6 grade II and 4 grade I meningiomas. Table 1 shows the detailed model validation results.

**Table 1:** Summary of detection performance using different networks.

	Pathologic Grade I (total 55)	Pathologic Grade II (total 41)		
Model type	ACR/SCR/TC_AB_/TC_A_/TC_B_	ACR/SCR/TC_AB_/TC_A_/TC_B_		
Predicted grade I	51/44/38/40/16	6/10/16/12/26		
Predicted grade II	4/11/17/15/39	35/31/25/29/15		

Model type	Accuracy	Sensitivity	Specificity	AUC
ACR	0.90 (0.80–1.00)	0.85 (0.67–1.00)	0.93 (0.75–1.00)	0.91
SCR	0.78 (0.70–1.00)	0.76 (0.57–1.00)	0.80 (0.75–1.00)	0.83
TC_AB_	0.66 (0.50–0.90)	0.61 (0.50–1.00)	0.69 (0.50–1.00)	0.73
TC_A_	0.72 (0.60–0.90)	0.71 (0.57–1.00)	0.73 (0.50–1.00)	0.79
TC_B_	0.32 (0–0.50)	0.37 (0–0.57)	0.29 (0–0.50)	0.45

ACR, SCR, TC_AB_, TC_A,_ and TC_B_ stand for asymmetric CNN with ratio (18:2), symmetric CNN with ratio (10:10), traditional CNN with T1-CE and T2-FLAIR, traditional CNN with T1-CE, and traditional CNN with T2-FLAIR, respectively.

**Figure 3:**
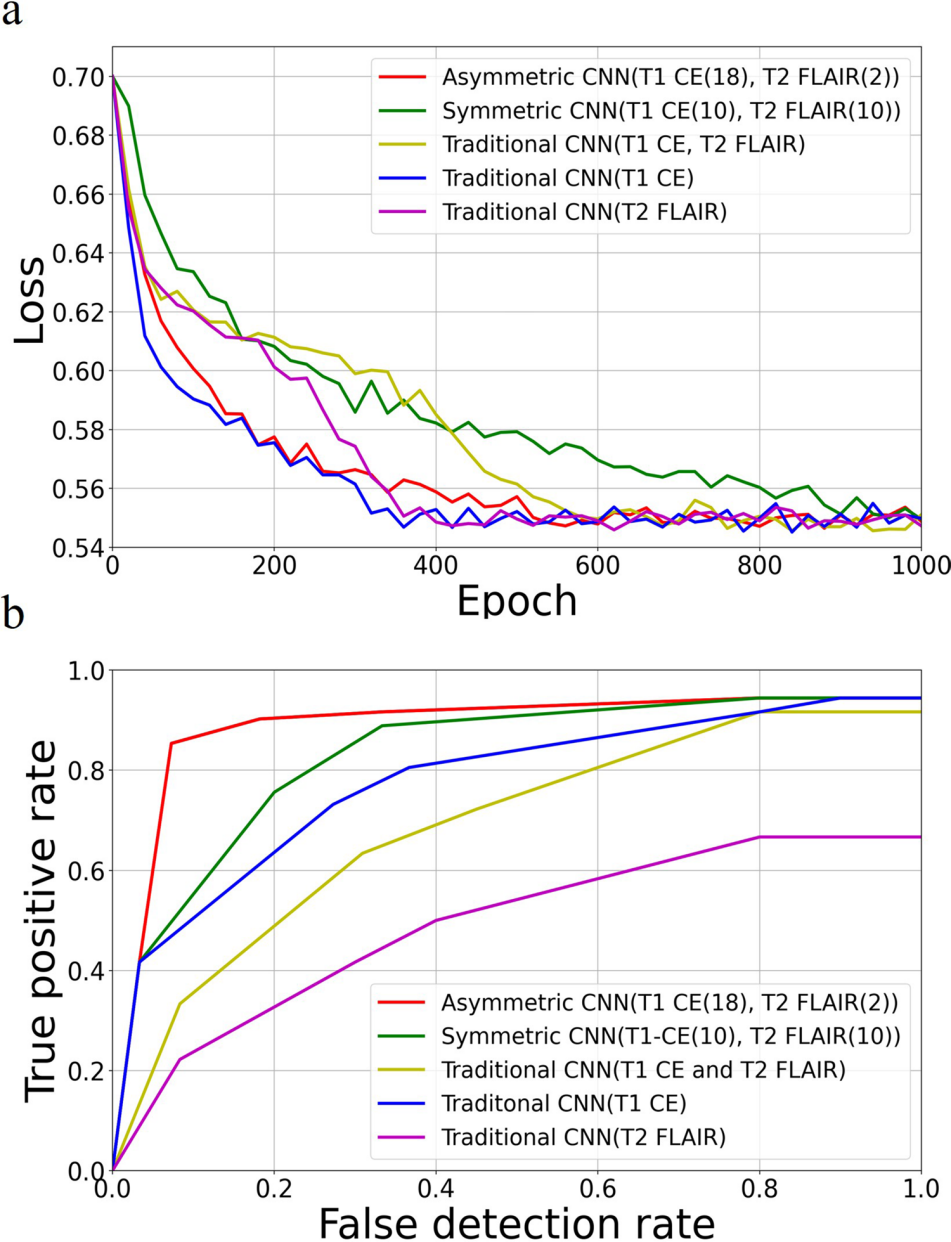
(**a**) The objective loss vs. epoch of the five types of CNN. (**b**) ROC curves from the five CNN models.

Figure 3a and b show the loss function convergence and the ROC for the five models, respectively. The proposed asymmetric CNN with a filter ratio of 18:2 for T1-CE vs. T2-FLAIR achieved the best classification performance. The area under the curve (AUC) values for different networks were 0.91 (asymmetric), 0.83 (symmetric), 0.73 (traditional with T1-CE and T2-FLAIR), 0.79 (traditional with T1-CE) and 0.45 (traditional with T2-FLAIR), respectively.

**Figure 4:**
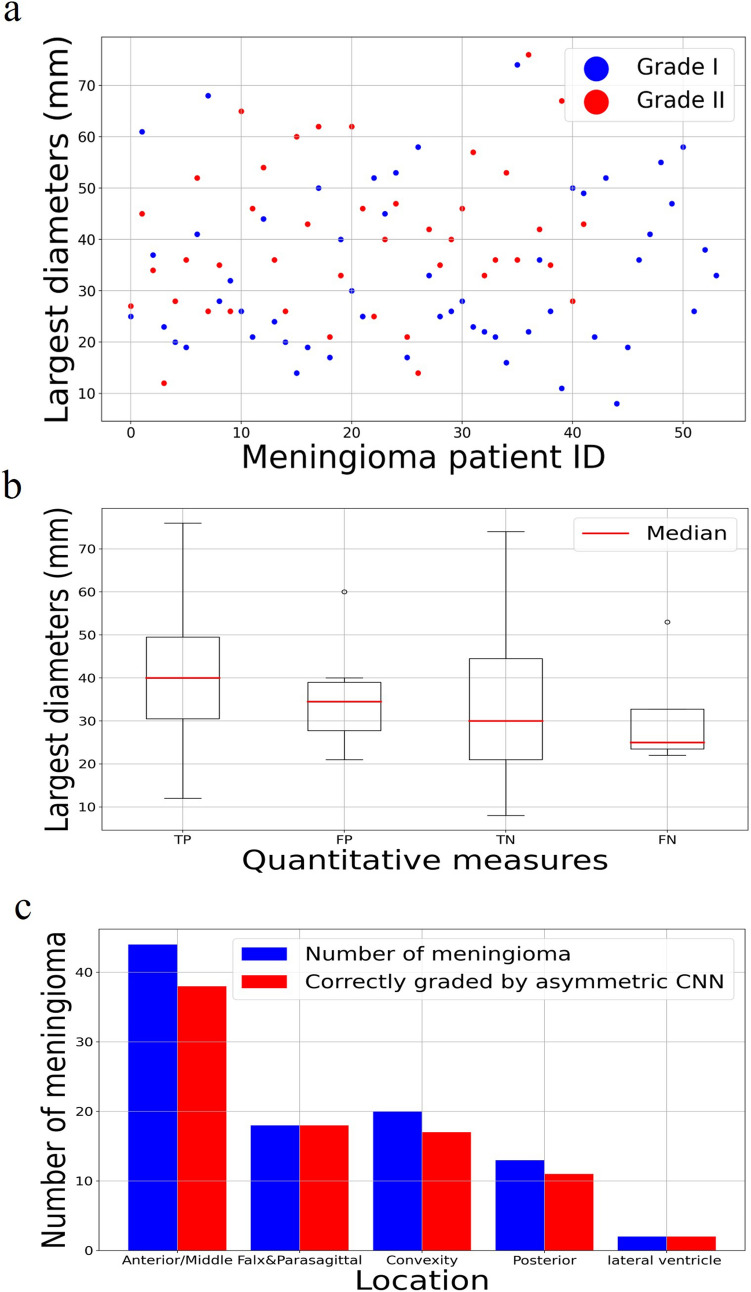
(**a**) The lesion size distribution by Grade. (**b**) Boxplots of lesion sizes for true positive (TN), false positive (FP), true negative (TN), and false negative (FN) grading. (**c**) Zonal distribution with correctly graded by asymmetric CNN.

Figure 4a shows the lesion size distribution by Grade, and there is no statistically significant difference (p = 0.03) between the Grade I and II groups. Figure 4b shows the lesion size distribution by classification results with no statistically significant difference (p = 0.03) observed. Correct grading based on location was further evaluated, as shown in Fig. 4c. The highest accuracy was seen in falx/parasagittal meningiomas at 1.0, while the lowest accuracy was seen in posterior cranial fossa meningiomas at 0.85. Small sample sizes are seen in each of these locations as most meningiomas in our cohort were located within the anterior and middle cranial fossa. The number of meningiomas per location, correctly predicted meningiomas, and accuracy was (43/38/0.89) for the anterior/middle cranial fossa, (18/18/1.0) for the falx/parasagittal, (20/17/0.85) for the convexity, and (13/11/0.85) for the posterior cranial fossa meningiomas. The dataset was also divided into two groups, supratentorial (40) and skull base/infratentorial (56), and the asymmetric CNN achieved an accuracy of 0.90 and 0.89, respectively. The ratio (R) of contoured volumes on T1-CE vs. T2-FLAIR ranged from 0.07 to 3.19 (mean ± std, 0.83 ± 0.50 ). Median R is 0.80 with an interquartile range of 0.54. The subtypes of meningiomas and grading accuracy are listed in the Appendix (eTable 1).

## Discussion

In this study, a novel dual-path 3D CNN architecture with an asymmetric weighting filter was developed to classify the meningioma grade automatically. The novel approach using separate paths and an asymmetric filter improved accuracy, sensitivity, specificity, and AUC. With the application of our model, the correct Grade was assigned to 90% of the surveyed meningiomas. Pre-operative grading may be helpful to guide meningioma management and help determine patients who may benefit from treatment rather than observation alone^3–5^.

The higher performance of our model is achieved by combined training using both T1-CE and T2-FLAIR images, the two most commonly acquired MR sequences for diagnosis. Our model performance is superior to previous studies using a single path deep learning network based on the T1-CE images alone^13,14^. Banzato et al.^15^ achieved a slightly higher classification accuracy on ADC images but substantially worse performance on T1-CE images. However, besides the less commonly available ADC, the study did not take advantage of combined image modality training, particularly T2-FLAIR, for further improved performance. In addition to the direct visualization of the abnormal tumor perfusion by T1-CE, T2-FLAIR may be particularly useful in cases of brain-invasive meningiomas due to its ability to discriminate edema from compression versus infiltrative disease^23,24^.

However, combining the T2-FLAIR with T1-CE for auto-classification is not straightforward. As shown, using both images in a single encoding path resulted in compromised performance. Equally weighting both images in the two separate paths of the symmetric network also suffers from low accuracy. Therefore, a contribution of the current study is the use of a novel asymmetric classification network design, where an asymmetric filter allows optimization of the weighting of T2-FLAIR images relative to T1-CE images. The reduced weighting may be understood as follows. T2-FLAIR hyperintensity in the setting of neoplasms is attributed to vasogenic edema. Only ten of our patients had meningiomas with brain invasion. Therefore, in most patients, the T2-FLAIR hyperintensity is attributable to parenchymal compression (type 1 edema) as opposed to infiltrative disease (type 2 edema)^24^. Since type 1 edema is associated with compression, it is more closely related to the size of a lesion versus the infiltrative nature and aggressiveness of the disease.

Moreover, Fig. 4a shows that both Grade I and II meningiomas have a wide range of volumes, and in our study, size did not correlate with Grade. The ratio (R) between the contoured volumes based on the T1-CE and T2-FLAIR images for six misclassified grade II meningiomas were 0.18, 0.19, 0.59, 0.61, 0.18, 0.34, respectively. The Rs were substantially lower than the mean R of 0.83. In other words, misclassified patients tend to have more expansive T2-FLAIR volumes that need to be unbiased in the network training with the optimized 18:2 filter ratio while maintaining the contribution of T2-FLAIR for classification.

Historically, the implementation of current histological criteria for meningioma grading resulted in an improved concordance of grade designation by pathologists. However, discordances between pathologists still occur. A recent prospective NRG Oncology trial involving a central histopathologic review found a discordance rate of 12.2% in diagnosing WHO grade II meningiomas^25^. Discrepancies are likely multifactorial and may include differences between institutions and differences in diagnoses rendered by general surgical pathologists and subspecialty trained neuropathologists. Current criteria for diagnosing grade II meningiomas also span a wide breadth of pathological features (e.g., 4–19 mitotic figures/10 HPFs). Pathologic evaluation is limited as sampling errors can occur due to only assessing a finite number of slides over a continuous tumor. However, in our study, over 90% of the meningiomas were entirely sampled, reducing the possibility of under-diagnosis. The limitations of pathologic evaluation further highlight the importance of radiologically graded tumors to assist in predicting the natural course of a tumor. Auto-differentiation of specific subtypes was not possible due to the limited sample size and the large majority of Grade I and II patients being meningothelial (87%) and atypical (95%), respectively. Only one of the other Grade I histologies was incorrectly graded (1 of 5 secretory) and one of the other grade II histologies (1 of 2 chordoid). We hope that with larger datasets, the use of radiologic grading may assist in the elucidation of meningiomas that have unique histological and/or molecular findings and may help provide additional prognostic information, particularly for specimens that satisfy minimal diagnostic criteria for a grade II designation. To help alleviate grading discrepancies in this study, meningioma grading was confirmed by two subspecialty trained neuropathologists and one neuropathology fellow.

Our study is limited by a small sample size from a single institution. As a result, our study used tenfold validation instead of independent training and testing sets. Use of ADC or other functional MRs such as dynamic susceptibility contrast (DSC) and dynamic contrast-enhanced (DCE) are likely to improve the accuracy of our model further and have been shown to differentiate microcystic meningioma from traditional Grade I nonmicrocystic meningioma or high-grade glioma^26^. However, these additional MRs were not included due to limited samples. The meningiomas were also manually contoured, which introduces variability in selecting the regions of interest for our algorithm. Still, a standardized auto segmentation method is not available yet to decrease this variability. Our study also used imaging at a single time point and did not consider the rate of growth, which has been previously shown to correlate with Grade^27^. However, our CNN architecture predicted Grade with 90% accuracy based on a single time point of imaging features. It can therefore help to predict for patients who can be safely observed.

## Conclusion

A novel asymmetric CNN model with two encoding paths was successfully constructed to automate meningioma grade classification. The model significantly improved the grading accuracy with the two most commonly used MR sequences compared to single-path models trained with one or both MR sequences.

## Supplementary Information

Supplementary Information.
