# Supplementary material for: Automatic differentiation of Grade I and II meningiomas on magnetic resonance image using an asymmetric convolutional neural network
Source: Sci Rep. 2022 Mar 9;12:3806. doi: 10.1038/s41598-022-07859-0 (PMC8907289; doi:10.1038/s41598-022-07859-0)
Supplement: Supplementary file 1 — Supplementary Information. [file 41598_2022_7859_MOESM1_ESM.docx]

**Appendix**

eTable 1. Histologic subtypes and accuracy of Grade auto-differentiation

| Histologies | Total | Incorrectly Graded (%) |
| --- | --- | --- |
| Meningothelial | 48 | 4 (8.3) |
| Secretory | 5 | 1 (20) |
| Fibrous | 1 | 0 (0) |
| Psammomatous | 1 | 0 (0) |
| Atypical | 39 | 4 (10.3) |
| Chordoid | 2 | 1 (50) |
